# MRSA infections in Norway: A study of the temporal evolution, 2006-2015

**DOI:** 10.1371/journal.pone.0179771

**Published:** 2017-06-22

**Authors:** Francesco Di Ruscio, Jørgen Vildershøj Bjørnholt, Truls Michael Leegaard, Aina E. Fossum Moen, Birgitte Freiesleben de Blasio

**Affiliations:** 1 Department of Infectious Disease Epidemiology, Division of Infectious Disease Control, Norwegian Institute of Public Health, Oslo, Norway; 2 Department of Biostatistics, Institute of Basic Medical Sciences, University of Oslo, Oslo, Norway; 3 Department of Microbiology and Infection Control, Akershus University Hospital, Lørenskog, Norway; 4 Institute of Clinical Medicine, University of Oslo, Oslo, Norway; 5 Department of Clinical Molecular Biology (EpiGen), Division of Medicine, Akershus University Hospital, Lørenskog, Norway; Universidade de Lisboa Faculdade de Medicina, PORTUGAL

## Abstract

**Background:**

Norway has one of the lowest prevalences of methicillin-resistant *Staphylococcus aureus* (MRSA) infections in the world. This study exploits the extensive data on MRSA infections in the Norwegian surveillance system to investigate the important factors defining the MRSA epidemiology.

**Methods:**

We performed a quasi-Poisson regression of the monthly notification rate (NR) of MRSA infections reported from January 2006 to December 2015, comparing the time trend among people with an immigrant vs. Norwegian background and domestic vs. imported infections, stratified by age groups.

**Findings:**

A total of 5289 MRSA infections were reported during the study period, of which 2255 (42·6%) were acquired in Norway, 1370 (25·9%) abroad, and 1664 (31·5%) with an unknown place of acquisition. Overall, the monthly NR increased significantly from 2006 to 2015 (+0·8% each month). The monthly increase in immigrants (+1·3%) was steeper than that in people with a Norwegian background (+0·6%). There was a significant growth (+0·4%) in the rate of domestically acquired infections, however, the NR of infections acquired abroad increased faster (+0·8%). For both imported and domestic infections, the increase occurred in persons aged < 70 years.

**Interpretation:**

Our analysis suggests that immigration and importation, especially among persons aged < 40 years, represent important factors for the increasing notification rate of MRSA infections in Norway.

## Introduction

The global spread of methicillin-resistant *Staphylococcus aureus* (MRSA) over the past 20 years has become a major worldwide public health concern [1,2]. In some areas of the world, MRSA prevalence is very high, for example, in Latin American countries prevalence is estimated to be >80% [3]. In other regions the prevalence is rising. Australia experienced an increase from 12% in 2000 to 19% in 2013 [4]. In India proportions of 41–80% were observed in 2008–2012 [3]. Although the mean prevalence of MRSA is decreasing in Europe, the United States and Canada, the prevalence of MRSA is still high in most countries, ranging from 15% to 45% [3]. In Norway, the Netherlands and Sweden, effective surveillance and infection control programs have helped keep the percentage of invasive *S*. *aureus* resistant to methicillin below 1% [5]. However, recent studies analyzing data until 2011 reported an increase in the incidence and prevalence of MRSA isolates in Norway, which suggests a change in MRSA epidemiology [6–8]. We hypothesize that increased international travel, in addition to increased immigration to Norway, may be important drivers for the spread of MRSA through the country and the rise of the prevalence.

The low endemic level combined with the detailed national registry data available in Norway, constitutes an optimal framework to investigate changes in MRSA epidemiology. In the present study we investigated the recent trends in MRSA infections by analyzing national registry data between 2006–2015, with a particular emphasis on characterizing the temporal patterns of domestic vs. imported infections, stratified by age, and by Norwegian vs. immigrant background. This information is crucial for understanding the origin of the current increase in MRSA incidence, and to design targeted intervention strategies.

## Materials and methods

### Data

Data on MRSA infections in Norway, notified between January 2006 and December 2015, were obtained from the Norwegian Surveillance System for Communicable Diseases (MSIS) [9]. This registry contains all laboratory confirmed cases of MRSA that, by law, must be reported to the MSIS at the Norwegian Institute of Public Health by both medical doctors and microbiological laboratories. For each case, the clinical information including age, gender, birthplace, ethnic background, place of MRSA acquisition and the date of sampling were reported. The information on the place of acquisition is reported by treating doctors and it is defined according to information collected as part of the clinical interview. For MRSA infections acquired abroad, the country in which the individual was infected and the reason for visiting abroad was specified.

We classified cases into three population categories based on ethnic background: “Norwegians”, defined as persons with a Norwegian background or those older than nine-years-old adopted from abroad; “Immigrants”, defined as persons registered either as immigrants, Norwegian born to immigrant parents, children under nine-years-old adopted from abroad, or persons who arrived in Norway for purposes of family reunification or to seek asylum; and “Unknown/Others”, which included those with an unknown status and foreign tourists. The definition of immigrant background used in this study is a broad definition that includes also second generation immigrants. Persons adopted from abroad <9 years of age have been defined as persons with an immigrant background, assuming that they were children arriving in Norway already infected. The age limit was chosen considering the lowest value of the age variable in the dataset, which is stratified by age groups of 9 years.

We sorted the cases into four groups split by age: “0–19”, “20–39”, “40–69” and “≥70” years. The place of MRSA acquisition, registered in the dataset, was used to classify the infections acquired in Norway, abroad or reported with unknown place of infection.

Duplicated cases, defined as infected persons reported with the same MRSA strains more than once within a year, were excluded in the analyses (5 duplicated cases were found).

Quarterly total population data, annual total population data stratified by 10-year age groups, and annual data reporting the number of persons with immigrant background registered in Norway were obtained from Statistics Norway’s website [10]. The annual data was based on reporting from the first of January for each year within the study period. We calculated the population with a Norwegian background by subtracting the population with an immigrant background from the total population at a given timepoint.

### Statistical analysis

We performed a time series analysis of the monthly notification rate (NR) per 100,000 people of MRSA infections. The mid-monthly population estimates were used as the denominator, and were obtained from the linear interpolation of the relevant demographic datasets.

The NR time series were analyzed using weighted quasi-Poisson regression models. Unlike the classical Poisson model, a quasi-Poisson regression can handle over-dispersion in the data by introducing a dispersion parameter and defining the variance as a linear function of the mean.

The regression models used in our study, which trace the evolution of the mean level of the NR, *μ*, as a function of time, *t*, in months, are

$$\log\left(\mu_{i}\right)=\alpha +\beta \cdot t_{i}$$ Model 1

$$\log\left(\mu_{i}\right)=\alpha +\beta \cdot t_{i}+\gamma \cdot month_{i}$$ Model 2

In the first part of the analysis we studied the NR for all MRSA infections in the dataset to obtain an overview of the temporal evolution of the NR in Norway. We then grouped the infected persons according to either an immigrant or Norwegian background, and fitted the two time series to investigate differences between the two groups. We used Model 1 for these analyses. In additional analyses, we modified Model 1 to include fixed covariates: international flights arriving in Norway and immigration rate; and ethnic background and place of acquisition with interaction terms. These analyses are presented in the supplement (S1 Fig and S2 Table).

In the second part of the study we divided the analyses according to the place of acquisition of MRSA to investigate the NR of infections acquired in Norway and abroad, hereinafter also referred to as domestic and imported infections, respectively, and MRSA infections registered with an unknown place of acquisition. For each of the three places of acquisition, we analyzed the time trend of the NR using Model 2. The month of notification, *month*, added as a factor, allowed us to quantify changes in specific months compared to January, the reference month. The analyses were repeated for each of the four age groups described above for each of the places of acquisition. For the fit of the time series, we used a backward elimination approach starting from the full model and manually removing the covariates that were not statistically significant (p-value>0.05). Model 1 was also used to compare the NR level in January 2006 and in December 2015 for all the analyzed times series.

The results obtained from the quasi-Poisson regressions are reported in S1 Table.

In addition to the time series analysis, we calculated the annual proportions of ethnic backgrounds of people infected with MRSA for each place of acquisition (Norway, abroad and unknown place of acquisition).

We also attempted to classify the infections with an unknown place of acquisition using the Random Forest method. However, since the obtained classification error was too high (almost 30%), we instead analyzed the “Unknown” cases as a third separate component that we compared to domestic and imported infections.

The statistical analyses were performed with R (version 3.1.2) [11]. The R package “ggplot2”[12] was used to visualize the results from the analysis.

The study was approved by the Regional Committees for Medical and Health Research Ethics—South East Norway (project number 2011/2456).

## Results

### Total picture

A total of 5289 MRSA infections were reported in Norway from January 2006 to December 2015 (Table 1). The majority were wound infections or skin abscesses (73.3%); 10.6% were other non-severe infections and 4.8% were infections in inner organs. The remaining proportion of cases were registered as unknown.

**Table 1:** Number of MRSA infections grouped by gender and age group.

	No. of cases	Gender (%)		Age groups (%)			
		F	M	0–19	20–39	40–69	≥70
**Total MRSA infections**							
**Period: 2006–2015**	5289	44.6*	55.2	23.1	31.3	29.4	16.2
**Year: 2006**	338	49.4	50.6	19.5	27.8	29.3	23.4
**Year: 2015**	786	42.0	58.0	26.5	31.7	28.7	13.1
**MRSA infections acquired in Norway**							
**Period: 2006–2015**	2255	48.7	51.3	22.9	24.4	27.2	25.4
**Year: 2006**	208	49.0	51.0	17.8	23.5	30.3	28.4
**Year: 2015**	279	45.9	54.1	24.7	24.0	29.7	21.5
**MRSA infections acquired abroad**							
**Period: 2006–2015**	1370	39.5†	60.1	24.4	38.8	30.6	6.2
**Year: 2006**	82	47.6	52.4	25.6	36.6	28.0	9.8
**Year: 2015**	204	33.8	66.2	28.9	37.8	28.4	4.9
**MRSA infections with an unknown place of acquisition**							
**Period: 2006–2015**	1664	43.3‡	56.3	22.4	34.2	31.6	11.8
**Year: 2006**	48	54.2	45.8	16.7	31.2	27.1	25.0
**Year: 2015**	303	43.9	56.1	26.4	34.6	28.0	11.0

For each analyzed scenario we report the MRSA infections notified in Norway during the entire study period (2006–2015), in 2006 only, and in 2015 only.

* 12 cases (0.2%) were reported with the variable gender undefined.

† 5 cases (0.4%) were reported with the variable gender undefined.

‡ 7 cases (0.4%) were reported with the variable gender undefined.

**Fig 1:**
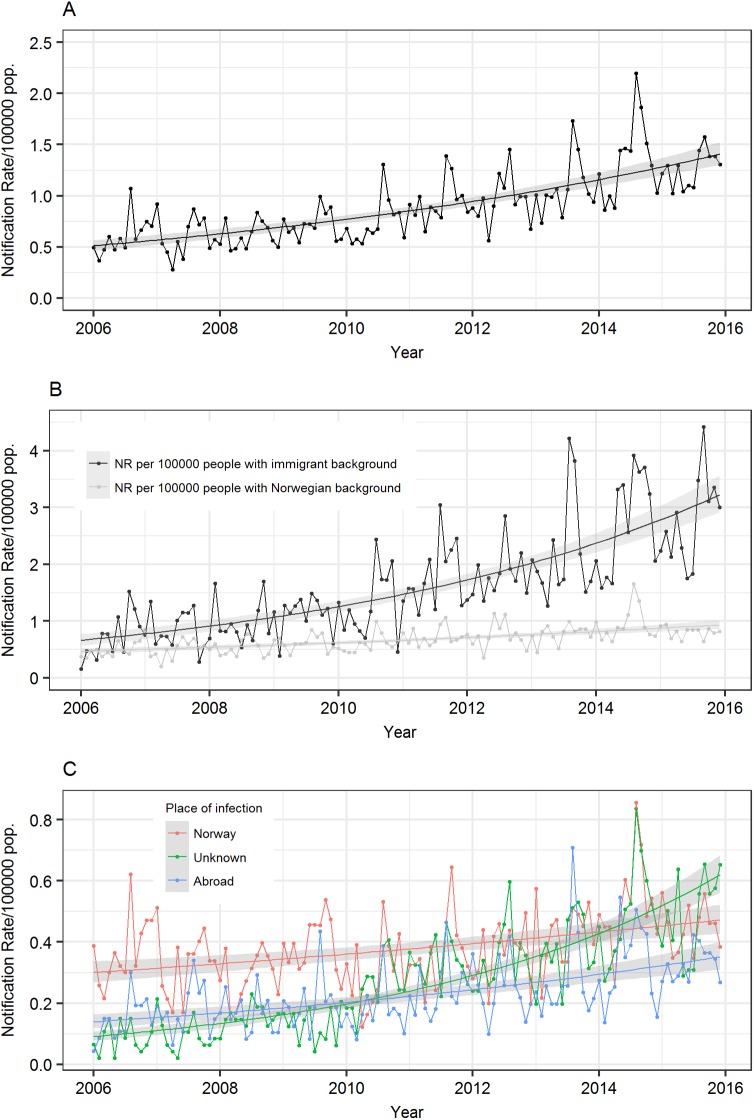
Time series of the monthly notification rate. (A) Monthly notification rate per 100,000 people for all MRSA infections registered in the Norwegian Surveillance System for Communicable Diseases between 2006–2015. The regression curve with the 95% confidence interval are represented. (B) Monthly notification rate per 100,000 people for MRSA cases with an immigrant or Norwegian background. For each time series the regression line and the 95% confidence interval are shown. (C) Monthly notification rate per 100,000 people of MRSA infections acquired in Norway (red), abroad (blue), and cases with an unknown place of infection (green). The regression lines are represented for each component with the 95% confidence interval in grey.

The mean level of the NR was estimated to increase by 0.8% per month, growing from NR = 0.51 in January 2006 to NR = 1.41 at the end of the study period (Fig 1A; Table 2), corresponding to 24 and 73 monthly cases, respectively. A significant association between the increasing trend (Fig 1A) and the total number of international flights arriving in Norway was found, with evident seasonal peaks in the summer period (S1 Fig).

**Table 2:** Temporal trends of the monthly notification rate (NR) per 100,000 population.

	% Monthly NR growth rate (95% CI)	NR mean level*	
		Jan 2006 (95% CI)	Dec 2015 (95% CI)
**Total MRSA infections**	0.8 (0.7–1.0)	0.51 (0.46–0.56)	1.41 (1.30–1.52)
**MRSA infections among cases with an immigrant background**	1.3 (1.1–1.5)	0.67 (0.55–0.79)	3.21 (2.90–3.52)
**MRSA infections among cases with a Norwegian background**	0.6 (0.45–0.72)	0.47 (0.42–0.52)	0.93 (0.85–1.01)
**MRSA infections acquired in Norway**			
**All ages**	0.4 (0.2–0.5)	0.30 (0.27–0.33)	0.47 (0.42–0.52)
**0–19 years**	0.7 (0.4–1.0)	0.21 (0.16–0.27)	0.53 (0.43–0.64)
**20–39 years**	0.4 (0.2–0.7)	0.27 (0.22–0.32)	0.45 (0.38–0.53)
**40–69 years**	0.3 (0.01–0.5)	0.23 (0.19–0.28)	0.32 (0.27–0.38)
**≥70 years**	Constant	0.92 (0.84–0.99)	0.92 (0.84–0.99)
**MRSA infections acquired abroad**			
**All ages**	0.8 (0.6–1.0)	0.14 (0.12–0.16)	0.35 (0.31–0.40)
**0–19 years**	1.1 (0.7–1.6)	0.10 (0.07–0.15)	0.41 (0.32–0.52)
**20–39 years**	0.9 (0.6–1.2)	0.18 (0.15–0.22)	0.55 (0.47–0.64)
**40–69 years**	0.6 (0.3–0.9)	0.13 (0.11–0.16)	0.26 (0.22–0.31)
**≥70 years**	Constant	0.13 (0.11–0.17)	0.13 (0.11–0.17)
**MRSA infections with unknown place of acquisition**			
**All ages**	1.6 (1.4–1.8)	0.10 (0.08–0.11)	0.62 (0.56–0.68)
**0–19 years**	1.7 (1.4–2.1)	0.07 (0.05–0.10)	0.59 (0.50–0.70)
**20–39 years**	1.8 (1.5–2.1)	0.10 (0.08–0.13)	0.85 (0.74–0.97)
**40–69 years**	1.6 (1.3–1.9)	0.08 (0.06–0.10)	0.51 (0.43–0.59)
**≥70 years**	1.0 (0.5–1.4)	0.17 (0.12–0.24)	0.52 (0.40–0.67)

Model-estimated temporal trends of the monthly notification rate per 100.000, by independent quasi-Poisson regressions on separate subsets of the data. The monthly changes, expressed as percentages, and the estimated mean levels of the NRs in January 2006 and December 2015 are reported for each analyzed scenario.

* To compare the NR mean levels, *μ*, in January 2006 and in December 2015 we used Model 1 (log(*μ*) = *α* + *β* ⋅ *t*).

We observed a significant monthly increase in the NR for persons with an immigrant background (+1.3%), and for those with a Norwegian background (+0.6%). At the end of the study period, the estimated level for persons with an immigrant background, NR = 3.21, was 3.4 times higher than that among persons with a Norwegian background, NR = 0.93 (Fig 1B; Table 2).

A significant increase of the NR was found among Norwegians acquiring infections in Norway (+0.3% each month; S2 Table). The growth of the NR was faster for infections with an unknown place of acquisition (+1.3% each month), but no significant differences were observed between infections acquired abroad and domestic cases (S2 Table; S2 Fig).

For persons with an immigrant background infected in Norway, the mean level of the NR in January 2006 was not significantly different from that of Norwegians. However, a significant difference was found in the time trends of the NRs, being steeper for the domestic infections among immigrants (+0.8% each month), compared to the domestic cases among Norwegians (S2 Table).

The interaction between ethnic background, place of acquisition and time was not statistically significant (S2 Table). Therefore, the difference in the monthly increase between persons with different ethnic backgrounds, observed for the domestic cases, was not affected when imported infections and infections with unknown place of acquisition were considered.

### MRSA infections acquired in Norway

In total, 2255 infections (42.6%) were acquired in Norway, with an almost equal proportion of men relative to women (Table 1). The mean level of the NR increased by 0.4% per month, arriving at NR = 0.47 in December 2015 (Fig 1C; Table 2), corresponding to 25 monthly cases.

Among domestic cases, 0–19 years-old children experienced the steepest NR increase (+0.7%), with a rise in the mean level from NR = 0.21 in January 2006 to NR = 0.53 in December 2015. The highest level, NR = 0.92, was observed in the ≥70 age group, which remained unchanged throughout the study period (Table 2).

We estimated a significant decrease of the NR between February and April, ranging between -23% and -39%, and in December (-25%), compared to January. The dip during the spring months was also significant in the notified infections among persons younger than 40 years of age (S1 Table).

**Fig 2:**
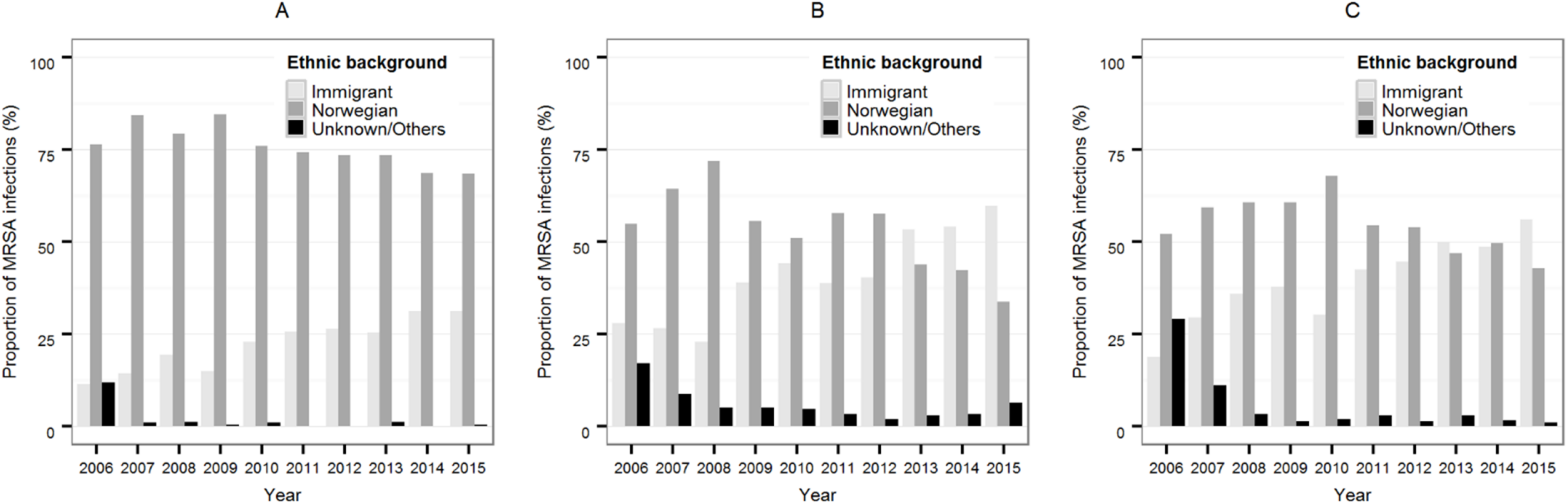
Annual proportions of MRSA infections by ethnic background. (A) MRSA infections acquired in Norway, (B) MRSA infections acquired abroad, and (C) MRSA infections reported with an unknown place of acquisition.

The proportion of infections in people with an immigrant background increased from 11.5% (24 cases) in 2006 to 31.2% (87 cases) in 2015, while in the same period the proportion of infected Norwegians decreased from 76.4% (159 cases) in 2006 to 68.5% (191 cases) in 2015 (Fig 2A).

### MRSA infections acquired abroad

During the study period, 1370 infections were acquired abroad (25.9%), mainly in Asia (43.3%) and Europe (24.9%) (Table 3). The NR increased by 0.8% each month, reaching in December 2015 a level of NR = 0.35 (Fig 1C; Table 2), corresponding to 18 monthly cases. NRs were significantly higher in August (+87%), compared to January (S1 Table).

**Table 3:** Distribution of the number of MRSA infections acquired abroad in the study period, 2006–2015.

Continent	Country	No. of cases	Gender (%)		Age groups (%)				Reason for being abroad (%)			
			F	M	0–19	20–39	40–69	≥70	Home visit	Infected before immigration	Study/Work/ Long-term stay/ business	Tourism
**Asia**		**593**	36.4*	**63.2**	**28.2**	**37.9**	**31.7**	**2.2**	**33.9**	**13.2**	**7.9**	**36.6**
	Philippines	185	31.3†	67.6	20.5	41.1	36.8	1.6	28.1	10.3	8.1	43.8
	Thailand	71	29.6	70.4	15.5	45.1	36.6	2.8	0.0	2.8	5.6	85.9
	India	57	49.1	50.9	21.0	43.9	35.1	0.0	47.4	5.3	10.5	24.6
	Sri Lanka	51	47.1	52.9	41.2	29.4	27.4	2.0	76.5	2.0	2.0	17.6
	Pakistan	40	42.5	57.5	35.0	22.5	32.5	10.0	90.0	5.0	2.5	2.5
**Europe**		**341**	**41.9‡**	**57.8**	**21.7**	**32.2**	**31.4**	**14.7**	**11.4**	**10.3**	**9.4**	**57.2**
	Spain	80	40.0	60.0	10.0	22.5	35.0	32.5	1.2	3.8	17.5	71.2
	Greece	48	52.1	47.9	27.1	25.0	37.5	10.4	0.0	2.1	2.1	93.8
	Turkey	26	46.1	53.9	42.3	23.0	27.0	7.7	7.7	0.0	0.0	84.6
	Poland	25	32.0	68.0	12.0	68.0	20.0	0.00	40.0	36.0	4.0	8.0
	Great Britain	16	43.7	56.3	18.7	31.3	31.3	18.7	0.0	18.8	31.2	25.0
**Africa**		**131**	**34.4**	**65.6**	**26.0**	**48.1**	**22.1**	**3.8**	**15.3**	**39.7**	**7.6**	**26.7**
	Egypt	24	33.3	66. 7	16.7	25.0	54.2	4.1	0.0	0.0	4.2	79.2
	Eritrea	21	33.3	66. 7	28.6	66.6	4.8	0.0	0.0	90.5	0.0	0.0
	Ethiopia	15	46.7	53.3	33.3	40.0	26.7	0.0	20.0	40.0	0.0	6.7
	Somalia	14	35.7	64.3	42.9	50.0	7.1	0.0	35.7	57.1	0.0	7.1
**Central and South America**		**101**	**48.5§**	**50.5**	**16.8**	**42.6**	**38.6**	**2.0**	**15.8**	**5.0**	**12.9**	**49.5**
	Brazil	31	41.9	58.1	25.8	35.5	35.5	3.2	9.7	6.5	29.0	38.7
	Cuba	24	33.3	66.7	12.5	25.0	62.5	0.0	20.8	4.2	4.2	66.7
**North America**		**81**	**51.9**	**48.1**	**18.5**	**43.2**	**24.7**	**13.6**	**4.9**	**16.0**	**16.0**	**43.2**
**Oceania**		**16**	**68.7**	**31.3**	**18.8**	**43.7**	**37.5**	**0.00**	**0.0**	**12.5**	**25.0**	**43.8**
**Unknown**		**107**	**32.7¶**	**66.4**	**22.4**	**45.8**	**28.0**	**3.8**	**0.0**	**41.1**	**10.3**	**20.6**

The countries with the highest percentage of cases acquired in their territories are reported for Asia, Europe, Africa, and Central and South America. Some of the notified cases have missing information in the dataset, thus some of the reported proportions do not add up to 100%.

* 2 cases (0.4% of the total) were reported with the variable gender undefined.

† 2 cases (1.1%) were reported with the variable gender undefined.

‡ 1 case (0.3%) was reported with the variable gender undefined.

§ 1 case (1.0%) was reported with the variable gender undefined.

¶ 1 case (0.9%) was reported with the variable gender undefined.

**Fig 3:**
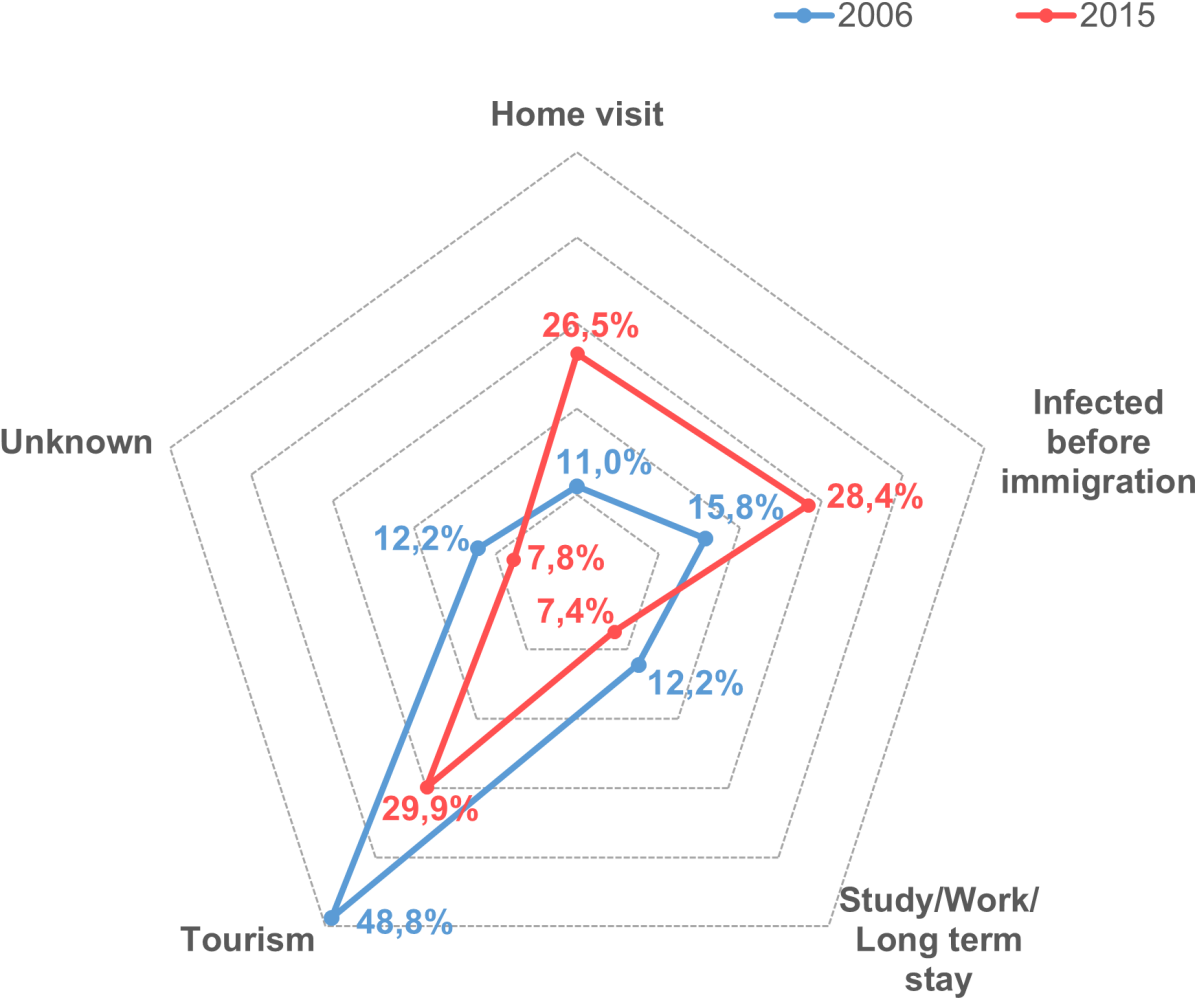
Graphical representation of the proportion of MRSA infections acquired abroad, grouped by the reason for being abroad. The blue line represents the proportions in 2006, and the red line represents the proportions in 2015.

In 2006, most of the imported infections were associated with tourism (48.8%), while 15.8% were related to immigrants arriving in Norway, and 11.0% were detected among persons who had visited family abroad. In 2015 the proportion of imported infections due to tourism had decreased markedly (29.9%), while the proportion of import due to family visits abroad had more than doubled (26.5%), and the proportion of immigrants arriving infected in Norway had increased to 28.4% (Fig 3).

The infections acquired in Asia were mainly associated with tourism (36.6%) and family visits (33.9%), and the infections from Europe were mostly acquired during tourist travel (57.2%).

Similar to domestic infections, the group 0–19 years experienced the highest monthly increase in NR (+1.1%). At the end of the period, the highest level of NR = 0.55, was observed among the group 20–39 years, and reflected a monthly increase of 0.9%. For the ≥70 age group, the level remained stable at NR = 0.13, and this was the lowest among the four age groups in December 2015 (Table 2).

The NR level for the youngest age groups were significantly higher in August, +221% for the 0–19 years of age and +60% for the 20–39 years of age, when compared to January (S1 Table).

We found that in 2006 54.9% (45 cases) were people with a Norwegian background and 28.0% (23 cases) were people with an immigrant background (Fig 2B). In 2015, the proportion of Norwegians had decreased to 33.8% (69 cases), and the proportion of cases with an immigrant background had risen to 59.8% (122 cases).

### MRSA infections with unknown place of acquisition

There were 1664 (31.5%) notified infections with an unknown place of acquisition (Table 1). The monthly growth of the NR (+1.6%) was steeper than the imported and the domestic infections, and the mean level changed from NR = 0.09 in January 2006 to NR = 0.62 in December 2015 (Fig 1C; Table 2), corresponding to 4 and 32 monthly cases, respectively.

There was a significant increase in the NR level in August relative to January (+51%), a pattern which was also observed among imported infections.

For people <70 years of age, the monthly increases of NRs were similar, ranging from +1.6 to +1.8%. The highest level of NR = 0.85 in December 2015 was observed among 20–39 years of age. Contrary to the time trends observed in domestic and imported cases, the NR of infections among persons ≥70 years of age increased (+1.0%) during the study period (Table 2).

For the 0–19 age group, significantly higher NR levels were observed in August (+130%), September (+91%), and November (+72%), relative to January. For the 20–39 age group, a similar pattern was observed in August (+53%) and September (+57%). For those over 70 years of age, the NR levels observed in May were decreased compared to January (-57%) (S1 Table).

The proportion of persons with a Norwegian background decreased from 52.1% (25 cases) in 2006 to 42.9% (130 cases) in 2015. Conversely, the proportion of persons with an immigrant background increased from 18.8% (9 cases) to 56.1% (170 cases) in that same period (Fig 2C).

## Discussion

Our results show that the monthly notification rate of MRSA infections in Norway has increased by a factor of almost three over the last ten years. The rise was particularly steep among people with an immigrant background, for whom the monthly increase is twice that of people with a Norwegian background. In December 2015, the mean NR among people with an immigrant background was 3.5 times higher than that among native Norwegians. The increasing trend was significant among both imported and domestic infections, but the rise in infections acquired abroad was steeper. The findings of this study suggest the emergence of new patterns in the MRSA epidemiology in Norway. Immigration and home visits appear to be increasingly important for importation of MRSA to Norway, while the import as a result of tourism has become less dominant. Most of the imported infections were acquired in Asia and Europe. Our analysis also highlights that the recent changes in MRSA epidemiology in Norway were largely driven by the younger age groups <40 years of age. For people over 70 years of age, the NR levels remained constant.

The observed increase in the reporting rate of MRSA infections is less likely to be explained by a change in the use of antimicrobials in Norway. Data on the use of antibiotics by user age, extracted from the Norwegian Prescription Database (NorPD) [13], show that in the last ten years the number of users per 1000 population of beta-lactam antibiotics has remained stable. Likewise, the prevalence of methicillin-susceptible *Staphylococcus aureus* (MSSA) infections has remained unaltered in the recent years [7].

Throughout the study period, individuals over 70 years of age, characterized by a constant trend, had the highest notification rate of the domestic infections. However, if the observed trends are to continue, within the next 5 to 10 years the NR level of the elderly will be matched by the increasing NR of people younger than 20 years of age. Our analysis corroborates the high level of the NR observed among elderly people in previous studies [8,14], but only for infections acquired domestically. Notably, in 2015, the number of people over 70 years of age with an MRSA infection acquired in Norway was almost three times higher than in 2006 (Table 1), and therefore, the observed rate is consistent with an aging population. Overall, the increasing number of infections found among domestic cases could be a consequence of an increasing endemicity of MRSA in Norway, which has also been suggested by Moxnes *et al*. [7], but other reasons should be explored.

We observed that the proportion of imported cases with an immigrant background increased between 2006 and 2015, and from 2013 importation from people with an immigrant background surpassed the proportion from native Norwegians. This may be a reflection of the rising number of immigrants arriving in Norway in recent years [15]. The effects of the increase of MRSA infections among persons with an immigrant background are also reflected in the data from 2015, which indicate that a larger number of people acquired infections while abroad visiting family, and an increase in the number of immigrants arriving in Norway already infected. This pattern is clearly different from that of 2006, where most of the imported infections were among people travelling abroad as tourists. Furthermore, infections acquired in Poland, the home land of the largest group of immigrant in Norway, and Pakistan, the country of origin of the largest group of immigrant from the Asian continent, are mainly associated with home visits. In the coming years, immigration may play an increasing role in the import of MRSA, especially with the migrant and refugee crisis which has faced Europe since 2015.

Our time series analysis of imported cases revealed that the strongest increase occurred within the age group 0–19. The analysis also highlighted a marked increase in the NR in August, a pattern which was only observed in individuals younger than 40 years of age. This peak may be explained by increased travel during the summer holidays. Our findings are consistent with recent studies that investigated how increased international travel facilitates the global spread of MRSA [16]. In the past decade the number of trips abroad for vacation increased from 4 million to 7.5 million [15], and the number of people who arrived in Norway on international flights increased by a factor of approximately 1.4 between 2009 and 2015. In particular, this number was found to have a significant effect on the trend of the monthly infections reported in Norway (S1 Fig).

Asia was the major source of imported infections, particularly the Philippines, a country where the proportion of MRSA in invasive *S*. *aureus* is 40–55% [3]. The large number of infections acquired in Asia may introduce new MRSA strains into Norway. For example, MRSA *spa* type t437, common in Asia [17], has been increasingly reported in Europe, especially in the north [18,19].

Spain was the source of the largest share of infections imported from within Europe, and was second only to the Philippines in imported infections to Norway. In contrast to the typical patterns of acquisition from other countries, however, a large proportion of the import from Spain involves elderly individuals. This may be a result of apartments and nursing homes for the elderly which had been built or purchased in Spain by some Norwegian municipalities.

Import of MRSA has also been documented in previous studies in Germany [20], Iceland [21], Norway [8], Denmark [22] and in a study including different European countries [23]. In Sweden, which has a low prevalence of MRSA, similar to Norway, an association was found between acquisition of MRSA and travel abroad and immigration [24,25]. Dissemination of MRSA in Norway following importation has also been suggested in previous studies that observed high heterogeneity of genetic lineages [26–29]. Internationally, another important driver of the MRSA epidemic is emerging: livestock associated (LA) MRSA. However, LA-MRSA is currently not commonly found in Norway [30].

The NR among infections with an unknown place of acquisition increased more rapidly than domestic and imported infections. By the end of 2015, the NR was almost twice that of imported cases and 1.3 times that of domestic cases. The significant peak observed in August, the age distribution of infected individuals, characterized by a higher number of young people, and the increasing annual proportion of people with an immigrant background, are all characteristics observed in the time series of infections acquired abroad. This could indicate that the majority of infections with unknown place of acquisition are imported.

A strength of this study is that we exclusively considered MRSA infections. The numbers of colonizations reported are prone to detection bias and will vary depending on the screening and contact tracing practices, which likely have changed during the study period, e.g. due to the revision of the national guidelines implemented in 2009 [31]. Here more focus was given to e.g. handling of MRSA-infected persons in health care services outside of hospitals as well as more specific recommendations on who to include in contact tracing activities. A strength of the study is also the combination of a time series analysis with the extensive Norwegian national data, which enabled us to quantify the evolution and the drivers of MRSA epidemiology over the last ten years in Norway.

On the other hand, it can be argued that the analysis of the impact of the importation vs. domestic circulation of MRSA was limited by lack of information on the place of acquisition, accounting for 31.5% of the total data. The increase of the proportion of infections with an unknown place of acquisition may be due to a genuine lack of information. However, it may also reflect an increasing negligence in reporting. Another limitation was the use of a quasi-Poisson model that assumes an exponential growth of the NR. Although the models were found to fit the data well, we did not test other functional forms to represent the time trends, such as linear ones. However, the model fits using an exponential vs. linear time trend would likely be only marginally different, as it has been found in another study [7], and we believe the choice of the functional form would not have affected the conclusions reached.

A clearer understanding of the drivers of the MRSA epidemic is critical for defining and implementing effective infection control measures. The quantitative role of immigration and travel abroad on the spread of MRSA infections in an increasingly globalized society needs further attention, especially in a country with a low MRSA prevalence and an increasing number of migrants and refugees. Our analyses suggest that changes in the composition of the infected population, and the rising proportion of cases with an unknown place of infection, are areas which need to be addressed by the future surveillance of MRSA in Norway. Changes in the epidemiology of resistant bacteria, such as MRSA, clearly indicate that it is now time for information earlier categorized as “nice to know”, such as the place of infection, to become “need to know”, and it is necessary to collate this information in order to be able to inform public health interventions.

## Supporting information

S1 Table Results obtained from the quasi-Poisson regression of the notification rate (NR) per 100,000 population for each different scenario considered in this study. For the time series analysis of MRSA infections acquired in Norway, abroad and with unknown place of infection, in addition to the intercept and the slope of the regression line, the months with a significant change of the NR level (p-value<0.05) are reported. The coefficients are expressed on the log-link scale.
(DOCX)

S2 Table Results obtained from the multivariable quasi-Poisson regression of the notification rate (NR) per 100,000 population with interaction terms between place of infection (POI), ethnic background (Bkg) and time (t). The reference level in the regression model is represented by Bkg = Norwegian background, POI = Norway and t = 0. The coefficients are reported in the log-link scale and they represent the variation compared to the reference level. The increase of the NR was significant among persons with a Norwegian background acquiring infections in Norway, *exp*(0.003) = 1.003 (i.e. +0.3% each month). Among Norwegians, the increase of the NR was faster for infections with an unknown place of acquisition, *exp*(0.003 + 0.01) = 1.013 (+1.3% each month); no significant differences were found between infections acquired abroad and domestic cases. A steeper increase was estimated among persons with an immigrant background acquiring infections in Norway, *exp*(0.003 + 0.005) = 1.008 (+0.8% each month), compared to Norwegians. The interaction term between POI, Bkg and t was not significant.
(DOCX)

S1 Fig Time series analysis of the monthly notification rate of MRSA infections. Monthly notification rate per 100,000 people (NR) for all MRSA infections acquired in Norway within 2006–2015. The red line represents the regression curve with the 95% confidence region in grey. The covariates of the model are time, immigration rate and number of international flights arriving in Norway (only available from 2009), obtained from the online database of Statistics Norway (SSB). A significant correlation was found between the NRs and the time series of international flights with a lag of one month. Thus, the time series of international flights has been shifted forward of one month. The increase factor was significant for time (*β*), 1.005 (95% CI 1.003; 1.009) for an increment of one month (p-value = 2.3 ⋅ 10^−5^) and for international flights (*δ*), 1.09 (95% CI 1.06; 1.13) for an increment of 100,000 flights (p-value = 7.15 ⋅ 10^−11^). The effect of the arrival rate of immigrants (*γ*) was not significant (p-value = 0.833).
(TIFF)

S2 Fig Time series of the monthly notification rate of MRSA infections. Monthly notification rate per 100,000 people (NR) for MRSA infections acquired within 2006–2015 in (A) Norway, (B) abroad and (C) in an unknown place. The NR for persons with a Norwegian and an immigrant background is reported for each place of acquisition.
(TIF)

S1 Dataset Number of MRSA infections reported in Norway. Monthly number of MRSA infections by ethnic background and place of infection reported in Norway in the period 2006–2015.
(XLSX)

## References

[1] Chambers HF, Deleo FR. Waves of resistance: Staphylococcus aureus in the antibiotic era. Nat Rev Microbiol 2009; 7: 629–41. doi: 10.1038/nrmicro2200 19680247 10.1038/nrmicro2200 PMC2871281

[2] Larsen AR, Bocher S, Stegger M, Goering R, Pallesen L V, Skov R. Epidemiology of European community-associated methicillin-resistant Staphylococcus aureus clonal complex 80 type IV strains isolated in Denmark from 1993 to 2004. J Clin Microbiol 2008; 46: 62–8. doi: 10.1128/JCM.01381-07 17989197 10.1128/JCM.01381-07 PMC2224276

[3] World Health Organization (WHO). Antimicrobial resistance: global report on surveillance 2014. WHO: Geneva; 2014.

[4] Center for Disease Dynamics, Economics & Policy (CDDEP). State of the World’s Antibiotics, 2015. CDDEP: Washington, D.C.; 2015.

[5] European Centre for Disease Prevention and Control (ECDC). Annual epidemiological report 2014. Antimicrobial resistance and healthcare-associated infections. ECDC: Stockholm; 2015.

[6] Moxnes JF, Moen AEF, Leegaard TM. Studying the time trend of Methicillin-resistant Staphylococcus aureus (MRSA) in Norway by use of non-stationary γ-Poisson distributions. BMJ Open 2015; 5: e007163 doi: 10.1136/bmjopen-2014-007163 26438133 10.1136/bmjopen-2014-007163 PMC4606436

[7] Moxnes JF, de Blasio BF, Leegaard TM, Moen AEF. Methicillin-resistant Staphylococcus aureus (MRSA) is increasing in Norway: a time series analysis of reported MRSA and methicillin-sensitive S. aureus cases, 1997–2010. PLoS One 2013; 8: e70499 doi: 10.1371/journal.pone.0070499 23936442 10.1371/journal.pone.0070499 PMC3731260

[8] Elstrøm P, Kacelnik O, Bruun T, Iversen B, Hauge SH, Aavitsland P. Meticillin-resistant Staphylococcus aureus in Norway, a low-incidence country, 2006–2010. J Hosp Infect 2012; 80: 36–40. doi: 10.1016/j.jhin.2011.10.004 22118858 10.1016/j.jhin.2011.10.004

[9] Norwegian Surveillance System for Communicable Diseases (MSIS). Norwegian Institute of Public Health [cited 2016 May 5]. http://www.msis.no/

[10] Statistics Norway (SSB) [cited 2016 Apr 19]. http://www.ssb.no/

[11] R Development Core Team. R: A language and environment for statistical computing. R Foundation for Statistical Computing, Vienna, Austria; 2008.

[12] Wickham H. ggplot2: Elegant Graphics for Data Analysis. Springer-Verlag New York, 2009.

[13] Norwegian Prescription Database (NorPD) [cited 2016 Jun 23]. http://www.norpd.no/

[14] Steens A, Eriksen HM, Blystad H. What are the most important infectious diseases among those ≥65 years: a comprehensive analysis on notifiable diseases, Norway, 1993–2011. BMC Infect Dis 2014; 14: 57 doi: 10.1186/1471-2334-14-57 24495775 10.1186/1471-2334-14-57 PMC3923236

[15] Kristiansen JE. This is Norway 2015. What the figures say. Statistisk sentralbyrå (SSB), Oslo/Kongsvinger; 2015.

[16] Zhou YP, Wilder-Smith A, Hsu LY. The role of international travel in the spread of methicillin-resistant Staphylococcus aureus. J Travel Med; 21: 272–81. doi: 10.1111/jtm.12133 24894491 10.1111/jtm.12133

[17] Song JH, Hsueh PR, Chung DR, et al Spread of methicillin-resistant Staphylococcus aureus between the community and the hospitals in Asian countries: an ANSORP study. J Antimicrob Chemother 2011; 66: 1061–9. doi: 10.1093/jac/dkr024 21393157 10.1093/jac/dkr024

[18] Glasner C, Pluister G, Westh H, et al Staphylococcus aureus spa type t437: identification of the most dominant community-associated clone from Asia across Europe. Clin Microbiol Infect 2015; 21: 163.e1–8. 25658555 10.1016/j.cmi.2014.09.010

[19] Rolo J, Miragaia M, Turlej-Rogacka A, et al High genetic diversity among community-associated Staphylococcus aureus in Europe: results from a multicenter study. PLoS One 2012; 7: e34768 doi: 10.1371/journal.pone.0034768 22558099 10.1371/journal.pone.0034768 PMC3338755

[20] Köck R, Mellmann A, Schaumburg F, Friedrich AW, Kipp F, Becker K. The epidemiology of methicillin-resistant Staphylococcus aureus (MRSA) in Germany. Dtsch Ärzteblatt Int 2011; 108: 761–7. 10.3238/arztebl.2011.0761 PMC3230165 22163252

[21] Köck R, Mellmann A, Schaumburg F, Friedrich AW, Kipp F, Becker K. The epidemiology of methicillin-resistant Staphylococcus aureus (MRSA) in Germany. Dtsch Ärzteblatt Int 2011; 108: 761–7. 10.3238/arztebl.2011.0761 PMC3230165 22163252

[22] Bartels MD, Boye K, Rhod Larsen A, Skov R, Westh H. Rapid increase of genetically diverse methicillin-resistant Staphylococcus aureus, Copenhagen, Denmark. Emerg Infect Dis 2007; 13: 1533–40. doi: 10.3201/eid1310.070503 18258003 10.3201/eid1310.070503 PMC2851516

[23] Bartels MD, Boye K, Rhod Larsen A, Skov R, Westh H. Rapid increase of genetically diverse methicillin-resistant Staphylococcus aureus, Copenhagen, Denmark. Emerg Infect Dis 2007; 13: 1533–40. doi: 10.3201/eid1310.070503 18258003 10.3201/eid1310.070503 PMC2851516

[24] Stenhem M, Ortqvist A, Ringberg H, et al Imported methicillin-resistant Staphylococcus aureus, Sweden. Emerg Infect Dis 2010; 16: 189–96. doi: 10.3201/eid1602.081655 20113546 10.3201/eid1602.081655 PMC2957988

[25] Larsson AK, Gustafsson E, Johansson PJH, Odenholt I, Petersson AC, Melander E. Epidemiology of MRSA in southern Sweden: strong relation to foreign country of origin, health care abroad and foreign travel. Eur J Clin Microbiol Infect Dis 2014; 33: 61–8. doi: 10.1007/s10096-013-1929-2 23922169 10.1007/s10096-013-1929-2

[26] Hanssen AM, Fossum A, Mikalsen J, Halvorsen DS, Bukholm G, Sollid JUE. Dissemination of community-acquired methicillin-resistant Staphylococcus aureus clones in northern Norway: sequence types 8 and 80 predominate. J Clin Microbiol 2005; 43: 2118–24. doi: 10.1128/JCM.43.5.2118-2124.2005 15872230 10.1128/JCM.43.5.2118-2124.2005 PMC1153739

[27] Fossum Moen AE, Tannaes TM, Leegaard TM. USA300 methicillin-resistant Staphylococcus aureus in Norway. APMIS 2013; 121: 1091–6. doi: 10.1111/apm.12077 23607421 10.1111/apm.12077

[28] Moen AEF, Storla DG, Bukholm G. Distribution of methicillin-resistant Staphylococcus aureus in a low-prevalence area. FEMS Immunol Med Microbiol 2010; 58: 374–80. doi: 10.1111/j.1574-695X.2009.00649.x 20459509 10.1111/j.1574-695X.2009.00649.x

[29] Monecke S, Aamot HV, Stieber B, Ruppelt A, Ehricht R. Characterization of PVL-positive MRSA from Norway. APMIS 2014; 122: 580–4. doi: 10.1111/apm.12181 24106794 10.1111/apm.12181

[30] NORM/NORM-VET 2014. Usage of Antimicrobial Agents and Occurrence of Antimicrobial Resistance in Norway. Tromsø/Oslo; 2015.

[31] National Institute of Public Health, The Norwegian Directorate of Health. [Infection control 16 MRSA-guidelines]. Nordberg Trykk AS, Oslo; 2009.

