# Supplementary material for: MRSA infections in Norway: A study of the temporal evolution, 2006-2015
Source: PLoS One. 2017 Jun 22;12(6):e0179771. doi: 10.1371/journal.pone.0179771 (PMC5480993; doi:10.1371/journal.pone.0179771)
Supplement: S1 Table — For the time series analysis of MRSA infections acquired in Norway, abroad and with unknown place of infection, in addition to the intercept and the slope of the regression line, the months with a significant change of the NR level (p-value<0.05) are reported. The coefficients are expressed on the log-link scale. (DOCX) [file pone.0179771.s001.docx]

**S1 Table**. **Results obtained from the quasi-Poisson regression of the notification rate (NR) per 100,000 population for each different scenario considered in this study.** For the time series analysis of MRSA infections acquired in Norway, abroad and with unknown place of infection, in addition to the intercept and the slope of the regression line, the months with a significant change of the NR level (p-value<0.05) are reported. The coefficients are expressed on the log-link scale

| Parameter | Estimate |  | 95% confidence interval | P-value * |
| --- | --- | --- | --- | --- |
| **Total MRSA infections [** $\boldsymbol{log(\mu)=\alpha+\beta\cdot t}$ **]** | | | | |
| Intercept (α) | -0.68 |  | -0.78; -0.57 | <0.0001 |
| Slope (β) † | 0.008 |  | 0.007; 0.010 | <0.0001 |
| Dispersion parameter ‡ | 2.65 |  | - | - |
| **NR of cases with an immigrant background [** $\boldsymbol{log(\mu)=\alpha+\beta\cdot t}$ **]** | | | | |
| Intercept (α) | -0.42 |  | -0.59; -0.25 | <0.0001 |
| Slope (β) | 0.013 |  | 0.011; 0.015 | <0.0001 |
| Dispersion parameter | 1.68 |  | - | - |
| **NR of cases with a Norwegian background [** $\boldsymbol{log(\mu)=\alpha+\beta\cdot t}$ **]** | | | | |
| Intercept (α) | -0.77 |  | -0.87; -0.67 | <0.0001 |
| Slope (β) | 0.006 |  | 0.004; 0.007 | <0.0001 |
| Dispersion parameter | 1.79 |  | - | - |
| **Parameter** | **Estimate** |  | **95% confidence interval** | **P-value** |
| **MRSA infections acquired in Norway: all ages [** $\boldsymbol{log(\mu)=\alpha+\beta\cdot t+\gamma\cdot month}$ **]** | | | | |
| Intercept (α) | -1.09 |  | -1.27; -0.93 | <0.0001 |
| Slope (β) | 0.004 |  | 0.002; 0.005 | <0.0001 |
| February (γ) § | -0.25 |  | -0.48; -0.03 | 0.028 |
| March (γ) | -0.23 |  | -0.45; -0.006 | 0.047 |
| April (γ) | -0.49 |  | -0.73; -0.25 | 0.0001 |
| December (γ) | -0.28 |  | -0.51; -0.06 | 0.015 |
| Dispersion parameter | 1.17 |  | - | - |
| **MRSA infections acquired in Norway: 0-19 years [** $\boldsymbol{log(\mu)=\alpha+\beta\cdot t+\gamma\cdot month}$ **]** | | | | |
| Intercept (α) | -1.41 |  | -1.81; -1.05 | <0.0001 |
| Slope (β) | 0.007 |  | 0.004; 0.010 | <0.0001 |
| April (γ) | -0.69 |  | -1.28; -0.14 | 0.02 |
| Dispersion parameter | 1.32 |  | - | - |
| **MRSA infections acquired in Norway: 20-39 years [** $\boldsymbol{log(\mu)=\alpha+\beta\cdot t+\gamma\cdot month}$ **]** | | | | |
| Intercept (α) | -1.15 |  | -1.46; -0.85 | <0.0001 |
| Slope (β) | 0.004 |  | 0.002; 0.007 | 0.001 |
| March (γ) | -0.58 |  | -1.03; -0.15 | 0.01 |
| April (γ) | -0.77 |  | -1.25; -0.31 | 0.002 |
| Dispersion parameter | 0.97 |  | - | - |
| **MRSA infections acquired in Norway: 40-69 years [** $\boldsymbol{log(\mu)=\alpha+\beta\cdot t}$ **]** | | | | |
| Intercept (α) | -1.45 |  | -1.65; -1.27 | <0.0001 |
| Slope (β) | 0.003 |  | 0.0001; 0.005 | 0.042 |
| Dispersion parameter | 1.25 |  | - | - |
| **MRSA infections acquired in Norway: ≥70 years [** $\boldsymbol{log(\mu)=\alpha}$ **]** | | | | |
| Intercept (α) | -0.09 |  | -0.17; -0.007 | 0.037 |
| Dispersion parameter | 0.99 |  | - | - |
| **Parameter** | **Estimate** |  | **95% confidence interval** | **P-value** |
| **MRSA infections acquired abroad: all ages [** $\boldsymbol{log(\mu)=\alpha+\beta\cdot t+\gamma\cdot month}$ **]** | | | | |
| Intercept (α) | -2.00 |  | -2.21; -1.79 | <0.0001 |
| Slope (β) | 0.008 |  | 0.006; 0.009 | <0.0001 |
| August (γ) | 0.63 |  | 0.40; 0.86 | <0.0001 |
| December (γ) | -0.31 |  | -0.60; -0.04 | 0.03 |
| Dispersion parameter | 0.95 |  | - | - |
| **MRSA infections acquired abroad: 0-19 years [** $\boldsymbol{log(\mu)=\alpha+\beta\cdot t+\gamma\cdot month}$ **]** | | | | |
| Intercept (α) | -2.45 |  | -2.95; -1.99 | <0.0001 |
| Slope (β) | 0.011 |  | 0.008; 0.014 | <0.0001 |
| August (γ) | 1.17 |  | 0.70; 1.67 | <0.0001 |
| October (γ) | 0.57 |  | 0.06; 1.11 | 0.04 |
| Dispersion parameter | 0.99 |  | - | - |
| **MRSA infections acquired abroad: 20-39 years [** $\boldsymbol{log(\mu)=\alpha+\beta\cdot t+\gamma\cdot month}$ **]** | | | | |
| Intercept (α) | -1.68 |  | -2.01; -1.36 | <0.0001 |
| Slope (β) | 0.009 |  | 0.007; 0.012 | <0.0001 |
| August (γ) | 0.47 |  | 0.12; 0.84 | 0.01 |
| **MRSA infections acquired abroad: 40-69 years [** $\boldsymbol{log(\mu)=\alpha+\beta\cdot t+\gamma\cdot month}$ **]** | | | | |
| Dispersion parameter | 0.90 |  | - | - |
| Intercept (α) | -1.89 |  | -2.26; -1.56 | <0.0001 |
| Slope (β) | 0.006 |  | 0.003; 0.008 | <0.0001 |
| December (γ) | -0.56 |  | -1.07; -0.08 | 0.03 |
| Dispersion parameter | 0.94 |  | - | - |
| **MRSA infections acquired abroad: ≥70 years [** $\boldsymbol{log(\mu)=\alpha}$ **]** | | | | |
| Intercept (α) | -2.00 |  | -2.22; -1.79 | <0.0001 |
| Dispersion parameter | 1.02 |  | - | - |
| **Parameter** | **Estimate** |  | **95% confidence interval** | **P-value** |
| **MRSA with an unknown place of infections: all ages [** $\boldsymbol{log(\mu)=\alpha+\beta\cdot t+\gamma\cdot month}$ **]** | | | | |
| Intercept (α) | -2.47 |  | -2.72; -2.22 | <0.0001 |
| Slope (β) | 0.016 |  | 0.014; 0.018 | <0.0001 |
| August (γ) | 0.41 |  | 0.15; 0.68 | 0.003 |
| Dispersion parameter | 1.36 |  | - | - |
| **MRSA with an unknown place of infections: 0-19 years [** $\boldsymbol{log(\mu)=\alpha+\beta\cdot t+\gamma\cdot month}$ **]** | | | | |
| Intercept (α) | -2.94 |  | -3.45; -2.47 | <0.0001 |
| Slope (β) | 0.017 |  | 0.014; 0.020 | <0.0001 |
| August (γ) | 0.83 |  | 0.35;1.36 | 0.001 |
| September (γ) | 0.65 |  | 0.15; 1.18 | 0.015 |
| November (γ) | 0.54 |  | 0.03; 1.08 | 0.043 |
| Dispersion parameter | 0.95 |  | - | - |
| **MRSA with an unknown place of infections: 20-39 years [** $\boldsymbol{log(\mu)=\alpha+\beta\cdot t+\gamma\cdot month}$ **]** | | | | |
| Intercept (α) | -2.49 |  | -2.89; -2.12 | < 0.0001 |
| Slope (β) | 0.018 |  | 0.015; 0.020 | < 0.0001 |
| August (γ) | 0.42 |  | 0.02; 0.84 | 0.05 |
| September (γ) | 0.45 |  | 0.05; 0.87 | 0.03 |
| **MRSA with an unknown place of infections: 40-69 years [** $\boldsymbol{log(\mu)=\alpha+\beta\cdot t}$ **]** | | | | |
| Dispersion parameter | 0.98 |  | - | - |
| Intercept (α) | -2.54 |  | -2.81; -2.29 | < 0.0001 |
| Slope (β) | 0.016 |  | 0.012; 0.019 | < 0.0001 |
| Dispersion parameter | 1.25 |  | - | - |
| **MRSA with an unknown place of infections: ≥70 years [** $\boldsymbol{log(\mu)=\alpha+\beta\cdot t+\gamma\cdot month}$ **]** | | | | |
| Intercept (α) | -1.45 |  | -1.99; -0.96 | < 0.0001 |
| Slope (β) | 0.010 |  | 0.005; 0.014 | < 0.0001 |
| May (γ) | -0.83 |  | -1.64; -0.10 | 0.034 |
| Dispersion parameter | 1.04 |  | - | - |
| * The p-values are computed using a z-test  † The slope of the model describes the trend of the NR. The exponentiated estimates of the slope give the monthly increase factor of the NR.  ‡ A value of the dispersion parameter >1 indicates over-dispersion in the data, i.e. the variance is larger than the mean value.  § The exponentiated estimates of the reported months represent the increase factor of the NR in those specific months compared to January, the reference level. | | | | |
