# Supplementary material for: MRSA infections in Norway: A study of the temporal evolution, 2006-2015
Source: PLoS One. 2017 Jun 22;12(6):e0179771. doi: 10.1371/journal.pone.0179771 (PMC5480993; doi:10.1371/journal.pone.0179771)
Supplement: S2 Table — The reference level in the regression model is represented by Bkg = Norwegian background, POI = Norway and t = 0. The coefficients are reported in the log-link scale and they represent the variation compared to the reference level. The increase of the NR was significant among persons with a Norwegian background acquiring infections in Norway, exp(0.003) = 1.003 (i.e. +0.3% each month). Among Norwegians, the increase of the NR was faster for infections with an unknown place of acquisition, exp(0.003 + 0.01) = 1.013 (+1.3% each month); no significant differences were found between infections acquired abroad and domestic cases. A steeper increase was estimated among persons with an immigrant background acquiring infections in Norway, exp(0.003 + 0.005) = 1.008 (+0.8% each month), compared to Norwegians. The interaction term between POI, Bkg and t was not significant. (DOCX) [file pone.0179771.s002.docx]

**S2 Table. Results obtained from a multivariable quasi-Poisson regression of the notification rate (NR) per 100,000 population with interaction terms between place of infection (POI), ethnic background (Bkg) and time (t).** The reference level in the regression model is represented by Bkg =Norwegian background, POI=Norway and t=0. The coefficients are reported in the log-link scale and they represent the variation compared to the reference level. The increase of the NR was significant among persons with a Norwegian background acquiring infections in Norway, exp(0.003)=1.003 (i.e. +0.3% each month). Among Norwegians, the increase of the NR was faster for infections with an unknown place of acquisition, exp(0.003+0.01)=1.013 (+1.3% each month); no significant differences were found between infections acquired abroad and domestic cases. A steeper increase was estimated among persons with an immigrant background acquiring infections in Norway, exp(0.003+0.005)=1.008 (+0.8% each month), compared to Norwegians. The interaction term between POI, Bkg and t was not significant.

| Parameter | Coefficients | 95% confidence interval | P-value |
| --- | --- | --- | --- |
| (Intercept) | -1.24 | -1.36; -1.12 | <0.0001 |
| Bkg: Immigrant background | -0.07 | -0.36; 0.21 | 0.6147 |
| POI: Abroad | -0.91 | -1.13; -0.69 | <0.0001 |
| POI: Unknown | -1.42 | -1.65; -1.19 | <0.0001 |
| t | 0.003 | 0.001; 0.005 | 0.0003 |
| Bkg: Immigrant background * POI: Abroad | 0.78 | 0.36; 1.21 | 0.0003 |
| Bkg: Immigrant background * POI: Unknown | 0.96 | 0.53; 1.40 | <0.0001 |
| Bkg: Immigrant background * t | 0.005 | 0.002; 0.009 | 0.0028 |
| POI: Abroad * t | 0.0003 | -0.0027; 0.0033 | 0.8310 |
| POI: Unknown * t | 0.01 | 0.008; 0.013 | <0.0001 |
| Bkg: Immigrant background * POI: Abroad * t | 0.0031 | -0.002; 0.008 | 0.2501 |
| Bkg: Immigrant background * POI: Unknown * t | -0.001 | -0.006; 0.004 | 0.7723 |
