# Supplementary figures and images for: MRSA infections in Norway: A study of the temporal evolution, 2006-2015
Source: PLoS One. 2017 Jun 22;12(6):e0179771. doi: 10.1371/journal.pone.0179771 (PMC5480993; doi:10.1371/journal.pone.0179771)

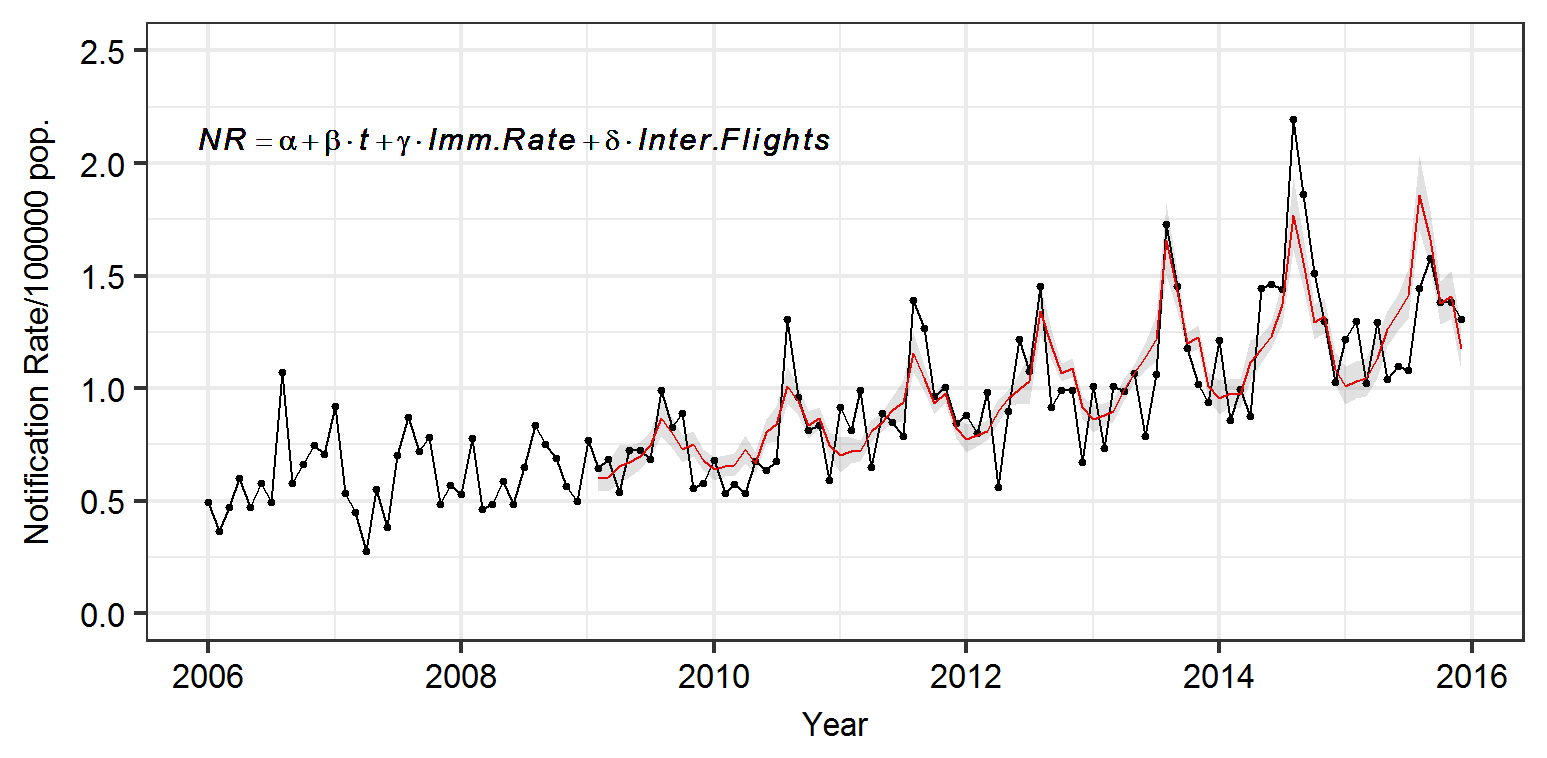

Supplement: S1 Fig — Monthly notification rate per 100,000 people (NR) for all MRSA infections acquired in Norway within 2006–2015. The red line represents the regression curve with the 95% confidence region in grey. The covariates of the model are time, immigration rate and number of international flights arriving in Norway (only available from 2009), obtained from the online database of Statistics Norway (SSB). A significant correlation was found between the NRs and the time series of international flights with a lag of one month. Thus, the time series of international flights has been shifted forward of one month. The increase factor was significant for time (β), 1.005 (95% CI 1.003; 1.009) for an increment of one month (p-value = 2.3 ⋅ 10−5) and for international flights (δ), 1.09 (95% CI 1.06; 1.13) for an increment of 100,000 flights (p-value = 7.15 ⋅ 10−11). The effect of the arrival rate of immigrants (γ) was not significant (p-value = 0.833). (TIFF) [file pone.0179771.s003.tiff]

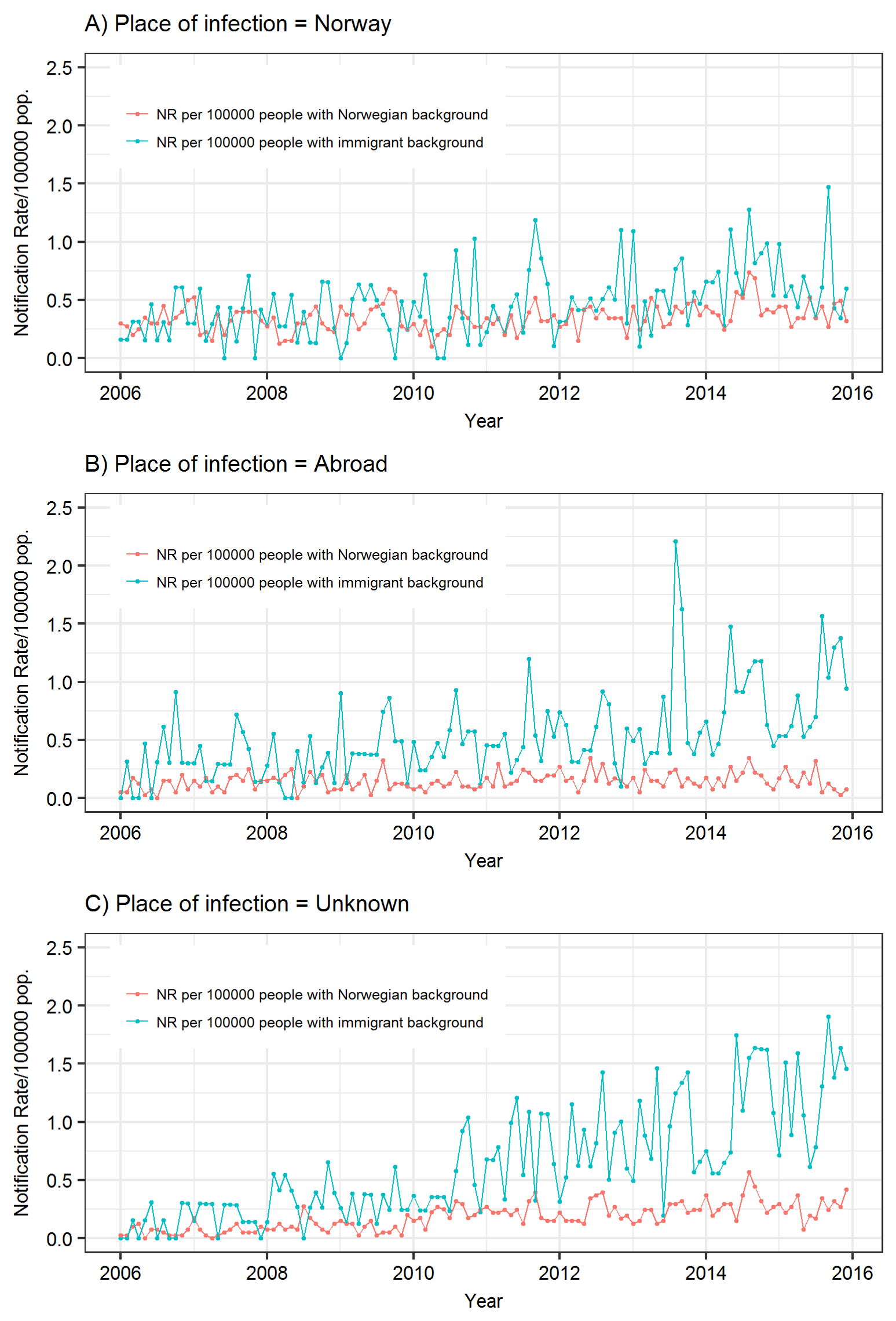

Supplement: S2 Fig — Monthly notification rate per 100,000 people (NR) for MRSA infections acquired within 2006–2015 in (A) Norway, (B) abroad and (C) in an unknown place. The NR for persons with a Norwegian and an immigrant background is reported for each place of acquisition. (TIF) [file pone.0179771.s004.tif]
